# Triple-Negative Breast Cancer Unveiled: Bridging Science, Treatment Strategy, and Economic Aspects

**DOI:** 10.3390/ijms26199714

**Published:** 2025-10-06

**Authors:** Valeriia Lebedeva, Mira Ebbinghaus, José Villacorta Hidalgo, Olaf Hardt, Rita Pfeifer

**Affiliations:** 1 Research and Development Department, Miltenyi Biotec B.V. & Co. KG, 51429 Bergisch Gladbach, Germany; valeriia.lebedeva@miltenyibiotec.de (V.L.); mira.ebbinghaus@miltenyibiotec.de (M.E.); josevillacorta.hidalgo@miltenyi.com (J.V.H.); olaf.hardt@miltenyibiotec.de (O.H.); 2 Medical Faculty, Rheinische Friedrich-Wilhelms-Universität Bonn, 53113 Bonn, Germany

**Keywords:** triple-negative breast cancer, targeted therapies, precision therapies, health care economics, health care access

## Abstract

Triple-negative breast cancer (TNBC) has historically been challenging to treat due to limited therapeutic options. Since 2018, the treatment landscape has evolved substantially with the approval of precision therapies, including immune checkpoint inhibitors, poly (ADP-ribose) polymerase inhibitors, and antibody–drug conjugates. Despite these advancements, the therapeutic benefit remains limited due to various clinical challenges, largely driven by TNBC heterogeneity and an incomplete understanding of drug–tumor crosstalk mechanisms—both contributing to a restricted pool of eligible patients and variable treatment responses. Concurrently, emerging drugs tested in recent pivotal TNBC trials have demonstrated variable outcomes. Additionally, the associated economic burden has become a pressing global concern, as several approved drugs provide insufficient clinical benefit in relation to high expenditures, often driven by drug pricing. The situation is particularly critical in low- and middle-income countries, where TNBC is highly prevalent, yet access to even chemotherapeutic treatment remains limited. These factors collectively hinder real-world patient outcomes. This review provides a comprehensive analysis of TNBC management, integrating clinical advancements with economic perspectives and raising awareness of underdiscussed topics. The overview presented herein highlights the necessity for a global, interdisciplinary approach and patient centered care in TNBC drug development.

## 1. Introduction

According to the GLOBOCAN report, female breast cancer became the most common type of malignancy in 2020 and was ranked among the five leading causes of cancer-related deaths worldwide [1]. Its global incidence is projected to rise by 38% by 2050, with annual deaths more than doubling, reaching 1.1 million [2].

Breast cancer is a heterogeneous disease with treatment strategies and prognosis varying by subtype. Clinically, it can be classified based on three markers: human epidermal growth factor receptor 2 (HER2) and hormone receptors (HR) for progesterone and estrogen. The expression levels are defined using immunohistochemistry (IHC), with (fluorescent) in situ hybridization (ISH) applied for HER2 when necessary. Thus, breast cancer is primarily categorized into three intrinsic subtypes: HR-positive, HER2-positive (HR-negative, HER2-positive), and triple-negative breast cancer (TNBC; HR- and HER2-negative) [3,4].

Comprising 10–20% of breast cancer cases, TNBC is considered the most aggressive subtype [5,6]. It is typically diagnosed at a younger age and more advanced tumor stage [7,8]. Until recently, its treatment development has lagged behind compared to other subtypes due to the lack of targetable markers, leaving TNBC patients with limited therapeutic options primarily centered on chemo- and radiotherapy. Consequently, patients with TNBC demonstrate significantly poorer outcomes than those with non-TNBC [6,9], with a 5-year overall survival (OS) of 64% versus 81% and 5-year progression-free survival (PFS) rates of 61% versus 70% in stage I-III disease [9]. Additionally, over 25% of TNBC patients experience recurrence [8,10], with 80% of those developing distant metastases [10], resulting in only 11–27% 5-year OS [6,11]. These poor outcomes prompted substantial efforts to better understand and develop novel therapeutic approaches. Consequently, since 2018, several precision drugs entered the TNBC drug market [12,13]. Despite these therapeutic advancements, several clinical challenges persist, including tumor heterogeneity, limited understanding of drug mechanisms and resistance pathways, and incomplete characterization of the disease biology. While these gaps are often the focus of translational research, the economic perspective remains heavily underdiscussed. However, the economic burden associated with TNBC has a profound impact on patients worldwide, particularly in developing and least developed countries. Even the standard-of-care treatments impose a substantial financial strain on patients, and novel treatment options can further exacerbate the expenses [14]. This review provides a comprehensive analysis of the current progress in TNBC drug development, examining both clinical opportunities for recently approved and emerging drugs, while highlighting the economic challenges within the TNBC drug market and patient community. By bridging this translational gap, we identify areas requiring enhanced cross-disciplinary coordination to improve TNBC patient-centered outcomes.

## 2. Approved Therapies

**Figure 1:**
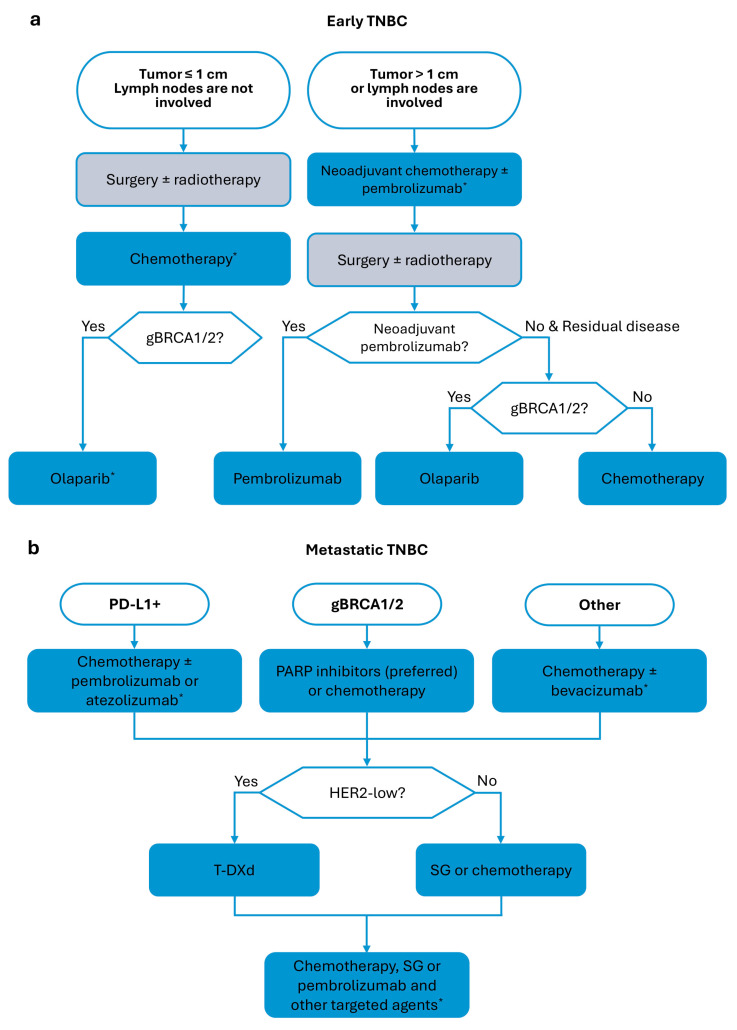
Simplified scheme of TNBC management, based on the European Society for Medical Oncology (ESMO) guidelines. (**a**) Management of early TNBC (adapted from Loibl et al., 2024 [13]). (**b**) Management of metastatic TNBC (adapted/updated from Gennari et al., 2021 [12]). Particular drugs (*) may be approved only in EU but not in US; approved only conditionally; used only with specific combinatorial therapy; and applicable only to a subset of patients.

The landscape of TNBC treatment has been transformed in recent years through the approval of precision drugs, including poly (ADP-ribose) polymerase (PARP) inhibitors (PARPis), immune checkpoint inhibitors (ICIs), antibody–drug conjugates (ADCs), and other targeted therapies, which have been incorporated into management plans alongside novel chemotherapeutic agents and their combinations (extensively reviewed previously [15,16]) (Figure 1). This chapter reviews the latest approvals of precision therapies and discusses the emerging clinical challenges associated with their implementation.

### 2.1. PARP Inhibitors

A subset of (TN)BC patients carry germline mutations in the breast cancer-associated gene 1 or 2 (gBRCA1/2), which normally function in DNA repair pathways. These mutations confer up to an 85% lifetime risk in developing breast cancer [17].

The therapeutic relevance of gBRCA1/2 was first demonstrated in two preclinical studies in the early 2000s, which showed that these mutations sensitize cells to PARP inhibition [18,19]. It prevents the repair of single-strand DNA breaks, resulting in the accumulation of double-strand breaks. In BRCA1/2-deficient cells, these breaks cannot be effectively repaired, causing cell death. More recently, other models of PARPi-induced synthetic lethality have been suggested, expanding our understanding of their therapeutic mechanisms [17].

Two pivotal phase III trials, OlympiAD (NCT02000622) and EMBRACA (NCT01945775), investigated the efficacy of two PARPis—olaparib (Lynparza; AstraZeneca and Merck & Co.) or talazoparib (Talzenna; Pfizer)—for patients with locally advanced (LA) and/or metastatic breast cancer harboring gBRCA1/2 mutations. Both drugs extended PFS by about 3 months when using olaparib (7.0 versus 4.2 months) or talazoparib (8.6 versus 5.6 months) compared to standard single-agent chemotherapy [20,21], leading to U.S. Food and Drug Administration (FDA) [22,23] and European Medicines Agency (EMA) [24,25] approvals. Notably, neither showed substantial improvement in median OS in both initial and long-term analyses [20,21,26,27]. However, extended analysis of OlympiAD trial indicated OS benefit when using olaparib as first-line treatment for gBRCA1/2 metastatic TNBC (mTNBC) [27]. Although the drugs have distinct on-/off-target profiles in vitro [28,29], retrospective comparisons revealed no significant difference in efficacy and overall safety between olaparib and talazoparib in gBRCA1/2 mTNBC [30,31].

Beyond advanced TNBC, significant progress has also been made in addressing earlier disease settings. The phase III OlympiA study (NCT02032823) investigated the efficacy of adjuvant olaparib in HER2-negative gBRCA1/2 early breast cancer (including TNBC) patients, who had previously received chemotherapy. The study demonstrated a significant prolongation of invasive and distant disease-free survival (DFS) compared to placebo [32]. A follow-up analysis showed significant improvement in 4-year OS in the olaparib group compared to placebo (3.4% difference) [33]. Therefore, olaparib was approved for the adjuvant treatment of patients with chemotherapy-pretreated, gBRCA1/2 early TNBC in both US and EU [34,35]. This expansion of intervention points has enhanced olaparib’s market position in TNBC treatment, giving it an advantage over talazoparib.

In summary, both PARPis have emerged as viable treatment options for gBRCA1/2 TNBC. However, only less than one-third of TNBC patients carry BRCA1/2 mutations [36,37] and more than 40% of gBRCA1/2 cancer patients exhibit either inherent or acquired resistance to PAPRis [38]. Another remarkable clinical challenge is poor or no improvement of OS with PARPis. These factors severely affect overall clinical efficacy of PARPi treatment in TNBC. Another challenge is gBRCA1/2 testing. Previously, it was reported that around half of the patients with gBRCA1/2 breast cancer do not undergo genetic testing due to strict clinical guidelines [39]. While updated recommendations extended the criteria for genetical testing eligibility, other related challenges lead to poor referral and uptake rates and remain to be solved [40]. Consequently, a substantial proportion of patients remain in need of both novel therapeutic options, as well as optimization of PARPi therapy.

### 2.2. Immune Checkpoint Inhibitors

Harnessing the immune system to fight cancer has been extensively explored over recent decades. Leading approaches include ICIs—monoclonal antibodies (mAbs) that disrupt signaling pathways suppressing immune responses. A notable example is the blockade of programmed cell death protein 1—programmed cell death 1 ligand (PD-1/PD-L1) signaling pathway. However, a therapeutic key prerequisite is immunogenicity within the tumor microenvironment (TME).

While breast cancer generally has a low tumor mutational burden (TMB) [41], certain molecular subtypes of TNBC display notable immunogenicity [42,43]. Moreover, TNBC stands out with the most pro-inflammatory profile in comparison to the other subtypes [44,45]. Collectively, this distinct immunological landscape presents a promising therapeutic opportunity for the application of ICIs.

Groundbreaking progress has been achieved with pembrolizumab (Keytruda; Merck & Co.), an anti-PD1 mAb, first evaluated with chemotherapy in the phase III KEYNOTE-355 trial (NCT02819518). The results showed acceptable tolerability and improved outcomes when using pembrolizumab with chemotherapy compared to the placebo chemotherapy group in patients with high PD-L1 expression (combined positive score (CPS) of ≥10). The second interim analysis reported a notable PFS improvement (9.7 versus 5.6 months) [46], and extended follow-up showed a median OS gain of nearly 7 months (23 versus 16.1 months) [47]. These results led to FDA approval of pembrolizumab in combination with chemotherapy for recurrent/mTNBC with PD-L1 CPS > 10 [48], followed by EMA approval in 2021 [49]. Notably, only about 20% of TNBC patients have PD-L1+ tumors (results from non-FDA-approved test) [50], limiting its broader use.

Later, pembrolizumab was investigated in early TNBC in combination with chemotherapy as a neoadjuvant treatment followed by pembrolizumab monotherapy as an adjuvant treatment. Such a strategy improved the 5-year OS and event-free survival (EFS) compared to neoadjuvant chemotherapy alone (86.6 versus 81.7% and 81.2 versus 72.2%, respectively) regardless of PD-L1 status. This led to the authorization of pembrolizumab in neo- and subsequent adjuvant treatment for high-risk early-stage TNBC [51,52].

While pembrolizumab showed strong efficacy, the trial outcomes of atezolizumab (Tecentriq, Genentech/Roche), a mAb-targeting PD-L1, were ambiguous, leading to varying clinical guidelines across countries. Initially, atezolizumab, in combination with nab-paclitaxel (nab-p), showed encouraging improvement in PFS compared to the placebo plus nab-p (7.16 versus 5.49 months) in the phase III IMpassion130 trial (NCT02425891) for patients with advanced TNBC [53]. This led to its inclusion into the FDA’s Accelerated Approval Program followed by the EMA approval. However, based on the results of the following IMpassion131 trial (NCT3125902), in which the combination of conventional paclitaxel with atezolizumab did not improve the outcomes significantly [54], Roche withdrew the ICI for the treatment of mTNBC in the US. In contrast, clinicians in the EU may still consider atezolizumab in combination with nab-p for the treatment of PD-L1+ inoperable advanced TNBC, according to the positive IMpassion130 trial results [55,56]. The discrepancies between these trials raised a critical question: what is the optimal chemotherapeutic backbone? (further discussed in Clinical Trial Landscape, Antibodies)

Both pembrolizumab and atezolizumab inhibit PD1/PD-L1 crosstalk; however, the necessity, sufficiency, and level of PD-L1 expression cut-off represent a challenge. Therefore, several systematic analyses have been performed to better characterize suitable patient cohorts for ICI treatment. One of them analyzed atezolizumab and pembrolizumab in both adjuvant and neoadjuvant treatment settings and showed a clear benefit of using these ICIs with a more pronounced advantage for the patient subgroup testing positive for PD-L1 [57]. Interestingly, another study found no strong correlation between pathological complete response (pCR) and PD-L1 expression [58]. Contrasting results were obtained by two other groups who argued that patients with PD-L1-negative tumors did not demonstrate any noteworthy improvements [59,60]. Notably, studies that were included in these retrospective analyses used different test systems for PD-L1 detection and different scoring algorithms. Therefore, reliable test systems, more comprehensive trials, and a deeper understanding of drug–tumor crosstalk are required to draw definitive conclusions.

Besides PD-L1 expression, other markers indicating general immunogenicity recently emerged in solid cancer management, including TNBC: microsatellite instability (MSI), DNA mismatch repair deficiency (dMMR), and TMB. Extensive investigation of these immunogenic characteristics led to their clinical significance as prognostic and response prediction parameters. Thus, an important milestone was achieved when the FDA granted accelerated and subsequently full approval of pembrolizumab for the treatment of unresectable or metastatic pre-treated MSI-high/dMMR solid tumors, including TNBC [61]. Final approval was granted following a pooled analysis of the KEYNOTE-051, KEYNOTE-158, and KEYNOTE-164 trials, which demonstrated an overall response rate (ORR) of 33%. Among the responding patients, over 10% achieved a complete response rate (CRR) and 77% experienced responses lasting 12 months or longer [62]. In contrast, EMA imposed restrictions on eligible tumor types, excluding TNBC from the list. Later, pembrolizumab was also approved by the FDA for TMB-high solid tumors, including TNBC [63].

Notably, pembrolizumab is not the only option for late-stage TNBC in the US. Dostarlimab-gxly (Jemperli, GlaxoSmithKline), another anti-PD-1 mAb, received accelerated FDA approval as the third and later lines of therapy for advanced dMMR solid tumors based on early-phase trial results (NCT02715284) [64].

Overall, ICIs dramatically expanded their role in TNBC treatment, giving hope to numerous patients from early to late stages. However, many biological questions remain unanswered, including the optimal chemotherapeutic regimen, patient cohort, as well as long-term data on immune adverse events and drug efficacy.

### 2.3. Antibody–Drug Conjugates

ADCs represent another promising treatment option, functioning as “targeted chemotherapy” by linking therapeutic agents to tumor-targeting mAbs. This approach overcomes the off-target toxicities that limit conventional chemotherapy [65]. Sacituzumab govitecan (SG; Trodelvy, Gilead Sciences, Inc.) was the first ADC approved for TNBC treatment, combining a topoisomerase I inhibitor (SN-38, govitecan) with a mAb targeting trophoblastic cell-surface antigen-2 (TROP2; sacituzumab). Numerous cancer types were shown to overexpress TROP2, while its presence on healthy cells is restricted [66]. Additionally, its overexpression correlates with an unfavorable prognosis in breast cancer [67].

The phase III ASCENT trial (NCT02574455) was designed to investigate SG in LA or pretreated mTNBC compared to the treatment of the physician’s choice (TPC). Overall, SG showed improvement in median PFS (5.6 versus 1.7 months) and OS (12.1 versus 6.7 months) as the second and subsequent line of therapy [68], although concerns regarding the trial design were raised [69]. Notably, TROP2 expression was heterogenous among enrolled patients, and the improvement in end points of the trial positively correlated with TROP2 expression levels. However, patients with TROP2-low tumors had numerical improvement as well, although its significance could not be determined due to low numbers of the respective patients enrolled [68]. Thus, FDA and EMA approved the use of SG in patients with pre-treated metastatic or recurrent unresectable TNBC without restrictions on TROP2 expression levels [70,71].

Another ADC whose role in TNBC has recently evolved is fam-trastuzumab deruxtecan-nxki (T-DXd; Enhertu, AstraZeneca and Daiichi Sankyo). T-DXd consists of a topoisomerase I inhibitor (deruxtecan) linked to a humanized anti-HER2 mAb (trastuzumab). Initial approval was granted for HER2-positive metastatic breast cancer, since trastuzumab was developed and implemented for this subtype. However, later T-DXd was also investigated in the DESTINY-Breast04 trial for previously treated HER2-low breast cancer regardless of HR status (NCT03734029). According to a standard pathological report, biopsy is considered to be HER2-negative if HER2 expression is described as IHC0, IHC1+, and IHC2+/ISH- [4]. The latter two cases (IHC1+ or IHC 2+/ISH-) were used for the HER2-low definition in the T-DXd trail, which makes it relevant for this subset of TNBC patients. The authors reported significant improvement in median PFS (9.9 versus 5.1 months) and OS (23.4 versus 16.8 months) for patients treated with T-DXd in comparison with TPC [72]. Based on these results, T-DXd received approvals from the FDA and EMA for patients with pre-treated recurrent unresectable or metastatic HER2 IHC1+ or IHC2+/ISH- breast cancer [73,74].

Although the DESTINY-Breast04 study has highlighted the clinical relevance of the HER2-low breast cancer subtype, experts do not yet support creating an independent indication due to trial design limitations and concerns about HER2 testing sensitivity [4]. Given the comprehensive discussions of HER2-low breast cancer in European and American guidelines and by Ivanova et al. [4,75,76], this topic will not be further reviewed herein.

Overall, SG and T-DXd have emerged as promising options for patients with advanced TNBC. However, the clinical experience with ADCs in TNBC is limited, as with other novel approaches. While patients with a broad range of target expression can benefit from SG and T-DXd, the simple addition of mAb (trastuzumab) to chemotherapy does not yield any benefit for patients with target-low tumors [77]. This highlights the presence of a complex, yet understudied mechanism of ADC therapeutic action. Related matter is the development of resistance to ADCs, as reviewed elsewhere [78]. Among others, several antigen-unrelated mechanisms were proposed, namely ADC processing and payload resistance.

### 2.4. Others

While PARPis, ICIs, and ADCs have gathered the most attention as precision therapies for TNBC, other options exist under specific conditions. One such targeted therapy is bevacizumab (Avastin, Genentech), a mAb targeting vascular endothelial growth factor A (VEGF-A), thereby inhibiting tumor angiogenesis. In the phase III trial E2100, bevacizumab combined with paclitaxel showed improved PFS (11.3 versus 5.8 months) and ORR (48.9% versus 22.2%) over paclitaxel alone in metastatic HER2-negative breast cancer [79], leading to accelerated FDA and EMA approval in 2007–2008 [80,81]. However, subsequent studies failed to replicate these benefits, prompting the FDA to withdraw approval [81,82,83], a decision that sparked public debate [84]. A meta-analysis of eight trials confirmed a reduced PFS compared to E2100 and no significant OS benefit [85]. Preclinical studies also suggested antiangiogenic agents like bevacizumab may promote tumor progression by inducing hypoxia in the TME [86,87]. Despite this, bevacizumab remains approved in the EU in combination with paclitaxel or capecitabine [83,88]. Nonetheless, its use declined in some EU countries, such as Austria, following the FDA’s withdrawal [89].

Several drugs can be considered for third-line or later treatment of solid tumors with specific biomarkers in patients who have exhausted other options. These drugs target kinases linked to genetic abnormalities. For instance, tumors with neurotrophic tyrosine receptor kinase (NTRK) fusions respond to tropomyosin receptor kinase inhibitors like larotrectinib (Vitrakvi, Bayer), entrectinib (Rozlytrek, Roche), and repotrectinib, which are approved in the US and conditionally approved in the EU for NTRK+ solid tumors. Patients with rearranged during transfection (RET) gene fusion may be treated with selpercatinib (Retevmo, Eli Lilly and Company)—an RET receptor tyrosine kinase inhibitor. However, both fusions are rare in TNBC.

Thus, although treatment options have expanded, TNBC treatment remains challenging, and optimal drug implementation is yet unclear. Most novel options are either limited to specific biomarkers affecting few patients or restricted to advanced disease. Consequently, chemotherapy alone remains widely used for TNBC, while newer treatments still lack the complete understanding of their mechanisms and resistance patterns.

## 3. Clinical Trial Landscape

To address the unmet needs in TNBC, clinical research has surged over the past decade. Drawing from interventional studies started between 2016 and 2024 and cataloged in clinicaltrials.gov, this chapter evaluates the evolving landscape of treatment opportunities for TNBC patients.

**Figure 2:**
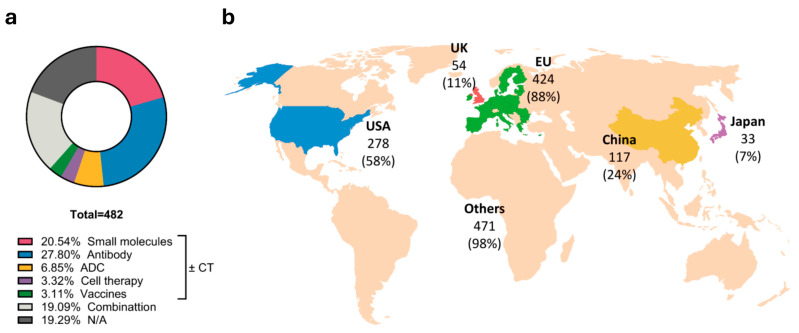
Overview of the interventional clinical trial landscape from 2016 to 2024. (**a**) Drug type distribution in the trials. (**b**) Geographical distribution of trial sites across five main pharmaceutical markets. The percentages represent the proportion of trials that include at least one center in the respective country relative to the total number of trials conducted worldwide.

For overview, the drugs used in the trials were categorized by antibodies (27.80% trials), small molecules (20.54% trials), ADCs (6.45% trials), cell therapies (3.32% trials), and vaccines (3.11% trials) (with or without chemotherapy (CT)) (Figure 2a). Additionally, 19.09% of trials investigate the combination of several precision drug entities (Figure 2a), with most of those studies focusing on small molecule–antibody combinations. Antibodies alone constitute the largest proportion, comprising monoclonal, polyclonal, bi-specific, and other types of antibodies. Small molecules comprise the second most studied approach, including PARPis, receptor agonists, cyclin-dependent kinase (CDK) inhibitors and others. In contrast, more innovative but high-risk approaches such as cell therapies and anti-cancer vaccines are less represented. Most trials are multi-centered and involve sites in several countries, with the EU being the most represented (Figure 2b). Among other areas with strongly developed pharmaceutical markets, the US has the highest number of trials, followed by China, the UK, and Japan (Figure 2b). The majority of trials include at least one center in countries beyond those listed above, with most of these sites located in Eurasia and North America. In contrast, only 70 from 482 trials (14.5%) have centers in Africa or Australia, despite the high incidence and/or mortality of TNBC in these regions [2]. The following sections highlight pivotal and contextually relevant trial designs and results (Table 1).

### 3.1. Small Molecules

Among small molecules, inhibitors of the phosphoinositide 3-kinase (PI3K) pathway (PI3K-Akt), a crucial cancer-related signaling cascade, have shown potential. Alterations in corresponding genes can drive cell proliferation, angiogenesis, and metabolic changes. Approximately one-third of TNBC patients exhibit increased PI3K-Akt activity [90], highlighting it as a therapeutic target. Following encouraging early-phase trial results, several PI3K-Akt inhibitors advanced to phase III trials. The novel pan-AKT inhibitor capivasertib (Truqap, AstraZeneca), which was approved by the FDA in 2023 for HR+/HER2- breast cancer, was also tested in combination with paclitaxel in a phase II trial for untreated mTNBC, demonstrating significant improvement in OS and PFS compared to the paclitaxel+placebo group, particularly for patients with tumor mutations in the targeted pathway (NCT02423603) [91]. However, preliminary results of the phase III CAPItello290 trial for LA or mTNBC (NCT03997123) were not satisfactory [92]. Similarly, another pan-AKT inhibitor, ipataserib (Roche), was investigated in combination with paclitaxel in the IPATunity130 trial (NCT03337724) and in combination with ICI (atezolizumab) and paclitaxel in the IPATunity170 trial (NCT04177108) for LA or mTNBC. However, both trials showed poor or no improvement in anti-tumor efficacy with either combination [93]. Another PI3K-targeting small molecule, alpelisib (Piqray, Novartis), was evaluated in the EPIK-B3 phase III trial in combination with nab-p (NCT04251533). The study had challenges due to slow recruitment, and primary end points were not met. The last drug targeting PI3K-Akt pathway identified in our search within phase III trials is everolimus (Afinitor, Novartis), an inhibitor of the mammalian target of rapamycin (mTOR), the latter kinase of the PI3K-Akt pathway. It is studied in combination with chemotherapy in a phase III trial (NCT05954442) for patients with luminal androgen receptor-positive (LAR) TNBC [43], which shows a higher frequency of PI3K-Akt pathway alterations than other TNBC molecular subtypes [94]. Although the androgen receptor is a compelling target in LAR TNBC, no targeted drugs have entered phase III evaluation. The current landscape of LAR TNBC was extensively reviewed elsewhere [95].

Important questions about PARPis currently investigated in clinical trials is the possibility of broadening their applicability. In patients with unselected early treatment-naïve disease (NCT02624973), olaparib achieved 56.3% ORR. BRCA1 methylation and mutations in genes involved in double-strand DNA break repair (e.g., gPALB2), occurring in approximately 80% of cases, have been identified as predictors of response [96]. However, the PARTNER trial (NCT03150576) showed that adding olaparib to chemotherapy did not improve the outcomes for gBRCA1/2 wild-type mTNBC [97]. Notably, the trials were not designed for direct comparison, and the decisive conclusion requires more investigation. Nevertheless, despite differing outcomes, both studies underscore the importance of thorough genetic testing.

Another key investigation in earlier clinical trials is the combination of PARPi with platinum-based chemotherapy. Given that TNBC is responsive to platinum agents, which share mechanistic similarities with PARPis, this raises questions about whether their effects are overlapping, synergistic, or potentially contribute to increased resistance. These issues were recently discussed in detail elsewhere [98,99].

Niraparib (Zejula, GlaxoSmithKline), approved by the FDA for certain cancers, is the only new PARPi currently under evaluation in a phase III trial (NCT04915755). Its ability to penetrate the blood–brain barrier [100,101] compared to olaparib and talazoparib may significantly improve the outcomes of TNBC patients with brain metastases.

Additionally, CDK 4/6 inhibitor trilaciclib (Cosela, G1 Therapeutics) was recently announced to fail to meet primary end points, though the official results have not been posted yet (NCT04799249). In China, a series of ongoing phase III trials is being conducted for famitinib (Jiangsu HengRui Medicine Co., Ltd.), a receptor tyrosine kinase inhibitor, in combination with chemotherapy and PD-1/PD-L1 ICI (NCT05760378, NCT05999149).

### 3.2. Antibodies

Antibodies represent another major drug class evaluated in nearly one-third of clinical trials investigating TNBC treatment either as a monotherapy or with chemotherapy. Approximately one-quarter of these trials are exploring the combination of antibodies with other novel approaches, such as PARPis or ADCs. While TNBC offers various antibody targets, most studies focus on blocking immunosuppressive pathways, primarily the PD-1/PD-L1 axis. Other checkpoints under early-phase investigation include CTLA-4, TIGIT, LAG-3, and CD39/CD73. Multi-targeting formats, such as bi- or tri-specific antibodies, are also being explored.

Agonist antibodies targeting immune cell activation receptors (e.g., 4-1BB, OX40) represent another strategy, though caution persists following the TeGenero incidence with the first-in-man trial of the super agonistic co-stimulation (CD28) antibody, whose administration led to life-threatening conditions in volunteers [102].

Most phase III trials concentrate on pembrolizumab or atezolizumab, as well as other PD-1/PD-L1 ICIs approved for other cancers, e.g., toripalimab (Loqtorz, Coherus BioSciences, Inc.). The TORCHLIGHT phase III trial (NCT04085276) showed promising preliminary results, where the combination of toripalimab with nab-p improved PFS for PD-L1+ advanced TNBC [103]. Another approved drug under investigation is avelumab (Bavencio, Merck and Pfizer). Preliminary results from its adjuvant therapy trial (NCT02926196) showed mixed outcomes: while OS was improved in the treatment cohort compared to observation, the primary endpoint of DFS was not achieved [104]. In addition, anti-PD-1 antibody camrelizumab (AiRuiKa, SHR-1210, Jiangsu Hengrui Medicine) is being investigated in multiple trials in China in combination with various drugs (NCT05760378, NCT05999149, and NCT06313463).

Despite pembrolizumab’s success in TNBC, several questions remain unanswered. One of them is the optimal chemotherapeutic backbone [105,106]. The TONIC trial (NCT02499367) investigated four different induction regimens prior to the administration of nivolumab (Opdivo, Bristol Myers Squibb), another anti-PD-1 mAb. While the trial was not designed for the direct comparison of treatment arms, it provided evidence in favor of doxorubicin pretreatment due to higher ORR, increased T-cell infiltration and T-cell receptor diversity in this arm [107]. The matter of chemotherapy choice to enhance ICI efficacy in TNBC was reviewed in detail by Giugliano et al. [108]. However, current evidence remains insufficient for clear conclusions and their implementation into clinical practice [108].

Another key area of investigation is ICI dosing. Unlike cytotoxic chemotherapy, where the maximum tolerated dose is expected to yield the best results, immunotherapy and, in particular, ICIs, operate differently and may be effective at lower doses. To explore this, the UNICANCER federation launched a multi-indication trial evaluating a reduced-dose ICI regimen (NCT05078047). If successful, this approach may not only reduce the risk of severe ICI-associated toxicities but also reduce the medication costs.

In contrast to ICIs, anti-angiogenic therapy with mAbs has shown limited efficacy, with bevacizumab, producing variable outcomes. Nevertheless, a bevacizumab biosimilar (BP102) is currently being evaluated in a phase III trial in China (NCT05806060) for the advanced TNBC of a basal-like immunosuppressed subtype [42]. Importantly, both mAbs selectively target VEGF-A, while other VEGF family members, such as VEGF-C and VEGF-D, are also expressed in breast cancer [109,110,111]. These factors can promote blood and lymphatic vessel formation by binding to VEGF receptors, thereby facilitating tumor progression [110,111]. Targeting these additional molecules, particularly through combinatorial approaches, may prove to be more clinically effective. Accordingly, several novel anti-angiogenic agents, mostly small molecules, are under investigation in early-phase clinical trials and have been comprehensively reviewed elsewhere [110,111].

### 3.3. ADCs

SG was initially approved by the FDA in 2020 as the first anti-TROP2 ADC. Since then, several trials have investigated SG with or without chemotherapy, especially in less pretreated disease (NCT05552001, NCT05382299). Two other TROP2-targeting ADCs in phase III TNBC trials are sacituzumab tirumotecan (ST; Merck & Co. under a license from Klus Pharma; NCT06279364, NCT05347134) and datopotamab deruxtecan (Dato-DXd; Datroway, AstraZeneca; NCT05374512).

As of January 2025, only one trial had reported results. In OptiTROP-Breast01 (NCT05347134), ST significantly outperformed chemotherapy in pre-treated advanced TNBC. It reduced the progression or death risk by 70%, improved PFS and OS, and demonstrated a tolerable safety profile [112]. Notably, patients were enrolled regardless of TROP2 expression [112], similar to the pivotal SG trial. These outcomes led to approval in China, though developments for the global market are still pending.

Beyond ADC monotherapy or in combination with chemotherapy, several trials are investigating ADC-ICI synergies. Among them, the ASCENT-04 trial (NCT05382286) evaluates a combination of SG with pembrolizumab as a first-line therapy for advanced PD-L1+ TNBC. The ASCENT-05 trial (NCT05633654) applies the same combination as post-surgical treatment for residual invasive disease. Similarly, AstraZeneca’s Dato-DXd is evaluated alone or in combination with durvalumab as a neoadjuvant or adjuvant treatment regimen (NCT05629585, NCT06112379).

Similarly to SG and T-DXd, ST and Dato-DXd deliver a topoisomerase I inhibitor. Given the potential for developing payload resistance [78,113] future research may focus on evaluating the efficacy of subsequent ADC application.

### 3.4. Others

Among ongoing phase III trials, the anti-Globo-H carbohydrate vaccine adagloxad simolenin/OBI-821 (NCT03562637) offers a novel approach for high-risk Globo-H-positive TNBC, developed by OBI Pharma [114]. Globo-H’s overexpression in tumors, including breast cancer, but limited presence in normal tissue, makes it a promising target for anti-tumor immunity. Another immune-boosting regimen Bria-IMT (engineered SV-BR-1-GM cell line, chemotherapy, and interferon) is undergoing evaluation in combination with ICI (NCT06072612) for mTNBC.

Despite the overall modest patient outcomes in pivotal clinical trials, many of these studies facilitate the generation of valuable translational data, offering insights into disease biology and tumor–drug interactions. These learnings are critical for informing the future drug development and optimizing clinical trial design.

## 4. Cost-Effectiveness of Approved TNBC Treatment Strategies

Several novel approaches are emerging in the TNBC treatment landscape, offering new hope for patients. In 2023, the market size (US, Japan, and five major European countries) was estimated at USD 3.1bn. Already approved drugs attempt to stretch the approval to earlier settings and some of the novel drugs under development may be approved within the next years. Therefore, the market is projected to grow up to USD 9.8bn by 2033 [115].

As the therapeutic options evolve, so do treatment costs and the associated economic burden. Most studies assessing it rely on data from before or shortly after the introduction of novel therapies. A systematic review analyzed 19 studies published between 2012 and 2021 [14], revealing significant cost variability but consistent trends. Compared to stage I-III TNBC, stage IV cases incurred 3–5 times higher costs, ranging from USD 100,000 to USD 300,000 (adjusted to 2021 U.S. dollars). Similarly, disease progression, recurrence, consecutive loss of productivity, and progress in therapy lines were linked to greater indirect costs. Direct cost analysis showed hospitalization as the primary driver, while systematic treatment dominated in short-term assessments.

While novel drugs are more expensive than standard chemotherapy, they can substantially reduce tumor burden, leading to a reduction in medical costs [14]. Therefore, investigating the cost-effectiveness of novel drugs is highly important. One crucial parameter is the quality-adjusted life year (QALY), which “weights life years lived by the quality of life experienced in each time period” [116]. Another widely used aspect is the incremental cost-effectiveness ratio (ICER), determined as “the difference in the expected costs of the two health technologies, divided by the difference in their expected effects (QALYs)” [116]. Comparing ICER to a specific value, e.g., willingness-to-pay (WTP) threshold (varies between countries) or the ICER of already available treatment, it is possible to conclude the cost-effectiveness of the drug [116].

Although studies are limited, cost-effectiveness data are available for most approved novel treatments. In Spain and Sweden, adjuvant olaparib for gBRCA1/2 high-risk early HER2-negative breast cancer was cost-effective compared to “watch and wait” and endocrine therapy [117,118,119], with ICERs of EUR 32,000–EUR 40,000 per QALY gained. Notably, these studies did not specify costs related to genetical testing for BRCA1/2 mutation detection. A separate analysis supported the cost-effectiveness of universal gBRCA1/2 testing for non-mTNBC in the US and China [120]. While olaparib showed clinical and economic benefits in early TNBC, outcomes for metastatic disease were less encouraging. Two studies concluded that gBRCA1/2 testing followed by olaparib had low cost-effectiveness in mTNBC [121,122], though extended testing for family members may improve the value [122]. Interestingly, findings on talazoparib in advanced disease were divergent. In the US, the costs for the talazoparib group were even lower than the ones for the standard therapy group, with a gain of 1.5 QALY [123]. Conversely, in Germany and Spain, talazoparib yielded a poor QALY gain (<0.5), with significantly higher costs compared to chemotherapy [124,125], raising concerns about its pricing and value.

Similarly, several studies concluded that there is a low probability of SG being cost-effective in the US and China [126,127,128]. SG costs were reported to have the most pronounced impact on respective expenses, highlighting the necessity of drug costs being significantly reduced. T-DXd, which is approved for HER2-low metastatic breast cancer, was shown to be cost-effective in Denmark [129] but not in the US [130]. Notably, T-DXd had a lower ICER in the TNBC subgroup (HR-negative, HER2-low) of the US study compared to the HR-positive subgroup [130].

Besides PARPis and ADCs, several studies estimated the cost-effectiveness of pembrolizumab and atezolizumab in various health care and patient settings. Analyses from the US, Switzerland, and Hong Kong concluded that neoadjuvant pembrolizumab with chemotherapy followed by adjuvant pembrolizumab is cost-effective for high-risk early TNBC [131,132,133]. Similarly, in the US, first-line pembrolizumab with chemotherapy was more cost-effective for mTNBC than atezolizumab with nab-p or chemotherapy alone [134]. In contrast, atezolizumab with nab-p, approved for advanced PD-L1+ TNBC in the EU and Japan, was found unlikely to be cost-effective in Singapore and Japan [135,136].

In summary, novel drugs, like pembrolizumab and olaparib, have shown cost-effectiveness in early-stage TNBC, supporting their integration in clinical practice. In contrast, analyses of talazoparib, SG, atezolizumab, and olaparib in mTNBC yielded mixed or unfavorable results. Only pembrolizumab combined with chemotherapy was cost-effective in metastatic disease. Several studies highlighted the possibility of enhancing the treatment value by decreasing the drug price, as it remains the major contributor to treatment expenses. Notably, the WTP threshold varied across studies, even within the same country. Most reported ICERs exceeded commonly cited WTP benchmarks of USD 50,000–USD 100,000 per QALY, highlighting the overall high cost of TNBC therapies. Therefore, more consistent, globally applicable strategies for cost-effectiveness evaluation and integration into drug development and clinical practice are urgently needed.

## 5. TNBC Pharmaceutical Market Challenges and Perspectives

TNBC is a complex disease with evolving treatments remaining insufficient for a cure. Beyond limited efficacy, concerns about the cost-effectiveness of new treatments reflect broader challenges in the TNBC drug market. Drug development is lengthy and expensive, with costs ranging from USD 648 million (2017 US dollars) [137] to USD 2.6 billion (2013 US dollars) [138], with additional expenses driven by post-approval costs [138]. A major cost driver is drug attrition—only 3.4% of anti-cancer candidates successfully progress from phase I to approval [139]. These factors lead to the current “high risk, high reward” market model, which affects drug prices and accessibility.

Rising economical expenses diminish the accessibility of drugs in both developed and low- or middle-income countries (LMIC). This is particularly critical in LMIC, where the incidence and burden of TNBC are disproportionately high [140,141,142,143]. An international study based on a physician questionnaire demonstrated a gap in the perception of most beneficial anti-cancer medications between developed countries and LMIC, as well as challenges in their affordability [144]. In LMIC, oncologists did not consider immunotherapy significantly beneficial for their patients, likely due to restricted access, though ICIs are established anti-cancer treatments. In contrast, half of high-income country respondents favored pembrolizumab (but not atezolizumab), despite often high out-of-pocket costs. Additionally, even essential cytotoxic medications like cisplatin and doxorubicin, commonly used for treating TNBC, remain unaffordable for LMIC patients, risking “catastrophic health expenditure”. A systematic review also found that poor affordability can lead to treatment refusal [145]. Thus, TNBC patients worldwide face significant economic barriers to care, with LMICs, where disease prevalence is highest, bearing the greatest burden.

Currently, the prices of modern drugs are not only high but also untransparent, raising concerns about their adequacy and substantiation. Therefore, global efforts are underway to improve affordability and stabilize pricing. A recent analysis showed that drug prices in the US have a limited correlation with clinical efficacy [146], echoing US and EU calls for value-based pricing [147,148]. In the US, Medicare was empowered to negotiate prices with manufacturers upon enactment of the inflation reduction act in 2022 [149], but its impact on cancer drugs may be limited due to the small number of eligible beneficiaries [150]. This limitation is especially problematic for TNBC, given the disease’s high heterogeneity. While these proactive measures are essential to reduce the economic burden, drug prices might decrease with the introduction of lower-cost biosimilars following patent expirations, or with the approval of currently developed drugs in competitive areas (e.g., PD-1/PD-L1 ICIs).

An important stakeholder of the drug market in the majority of developed countries is the health insurance provider. For TNBC patients, time to reimbursement (TTR) is particularly critical, as treatment costs rise with disease progression and TTR delays can worsen financial strain, especially for costly therapies like ICIs and ADCs. Strikingly, TNBC patients not only have high recurrence rates but also specific relapse kinetics—most relapses occur within 3 years after diagnosis [10]. Therefore, TNBC patients are particularly in need of timely help. However, in the EU, only Germany met the 180-day reimbursement deadline for anti-cancer medications, with compliance in other EU countries falling below 50% and TTR extending beyond 2000 days (median 407 days) [151]. Additionally, novel therapies often require expensive tests for biomarkers, e.g., BRCA1/2 testing for PARPi prescription. However, its timely incorporation into practice and insurance coverage are particularly poor.

While improvements in high-income countries can indirectly benefit LMIC, area-specific suggestions are also made to address drug market challenges. Key measures include expanding universal health care coverage, increasing cancer drug reimbursement, improving access to specialists and cancer centers, and enhancing screening capacities and diagnostic technologies [152,153].

## 6. Conclusions

TNBC is a subtype of breast cancer that is characterized by high mortality rates. Its historically poor prognosis stems from a lack of targetable biomarkers, limiting treatment options compared to other subtypes. Until recently, patients relied primarily on chemotherapy, with a five-year OS rate of 64% [9].

Despite substantial scientific progress leading to the approval of novel precision therapies, significant challenges remain. Most notably, the absolute improvements in patient outcomes have often been modest, raising the question of whether trial design and analysis have meaningfully advanced since the era of “universal” treatment.

To address this, ongoing clinical trials are exploring multiple strategies, including therapeutic combinations and the optimization of chemotherapy backbones. Additionally, novel treatments are under investigation in pivotal trials, including but not limited to small molecules mostly targeting the PI3K-Akt pathway, ICIs mostly against PD-1/PD-L1, and ADCs utilizing similar targets and payloads as SGs. While these investigational therapies offer hope, particularly for patients ineligible for approved drugs or those experiencing relapse, their ultimate impact remains uncertain, highlighting the gap between precision medicine theory and clinical reality. Many patients exhaust targeted options quickly, and emerging subclassifications in TNBC (e.g., HER-2-low TNBC) continue to complicate treatment decisions. From a development perspective, these limitations represent opportunities for innovation. Artificial Intelligence (AI)-driven approaches, adaptive trial designs, and real-world evidence integration hold promise to bridge this precision–benefit gap by enabling a more dynamic treatment selection and broader patient inclusion.

In this context, treatment strategies must be guided by a strong biological rationale. Additive approaches in clinical trials are justified only when they ensure scientific integrity and patient safety rather than simply increasing therapeutic intensity. Equally important are subtractive strategies, such as treatment de-escalation, which aim to reduce toxicity and cost. Though the latter is often underexplored due to limited commercial incentives, these approaches could be increasingly supported by non-profit institutions and in regions prioritizing cost-effectiveness. Future research must strike a balance between improving survival and minimizing treatment burden, with patient-centered outcomes and biomarker-driven trial designs at the core.

Despite the surge in new drugs and clinical trials for TNBC, economic considerations highlight that clinical success alone does not guarantee widespread patient access or commercial viability. Many therapies effective in early TNBC show low cost-effectiveness in metastatic disease. Additional barriers include the “high risk, high reward” drug market model and untransparent drug pricing, which are often misaligned from the actual therapeutic value, making these treatments financially inaccessible for many patients globally.

To achieve genuine improvements in TNBC patient outcomes, several key lessons must be integrated into modern drug development. First of all, TNBC is highly heterogeneous, and predicting responses to precision therapies remains a significant challenge. Therefore, a deeper understanding of mechanisms underlying the drug–tumor crosstalk, as well as of the disease itself, is essential. While TNBC has long been considered to lack biomarkers, recent findings derived from clinical studies suggest that, not only is it necessary for relatively “novel” biomarkers like PD-L1, but an even deeper profiling of the “conservative” HER2-negative status may be relevant for treatment strategy. These changes suggest a new era in (TN)BC management and drug development, with a closer focus on comprehensive personalized molecular profiling, moving beyond the classical three-subtype classification. However, it is important to note that the implementation of scientific knowledge into clinical practice should be mindful. While clinical guidelines must take up relevant, high-level evidence, their main role is to ensure standardized, safe, and effective patient care.

Moreover, economic considerations must be elevated to the same level of priority as clinical outcomes, as financial burden can severely impact patient medical decisions, in particular in countries with underdeveloped insurance coverage. High attrition rates across all stages of drug development contribute to the escalating treatment costs in oncology, especially in heterogeneous diseases like TNBC. Addressing these inefficiencies can improve both the affordability and accessibility of novel therapies.

Ultimately, these lessons can be summarized into one central principle of (TNBC) drug development: sustainable progress in patient-centered outcomes requires collaboration rather than competition between researchers, clinicians, industry, and regulatory authorities. Though long recognized, this principle is often overlooked, limiting its potential. Without such synergy, it is impossible to develop anti-cancer therapies that do not push patients to the brink of both biological and financial toxicity. The current period—while awaiting pivotal trial results—offers a strategic window to close the translational gap and realign efforts towards truly patient-centered innovation.

## Figures and Tables

**Table 1:** Overview of the design and outcomes of the recent phase III and relevant phase II TNBC trials. Inclusion criteria of “advanced” TNBC include locally advanced unresectable and/or locally recurrent and/or metastatic disease; #—number, ns—non-significant.

NCT #	Phase	Treatment Arms							Therapeutic Mechanism/ Target	Inclusive TNBC Stage and Biomarkers	Primary Endpoint(s), Results	
**Small molecules**												
NCT02423603	2	capivasertib paclitaxel				placebo paclitaxel			PI3K-Akt inhibition (pan-AKT) chemotherapy	advanced TNBC	PFS	
NCT03997123	3	capivasertib paclitaxel				placebo paclitaxel			PI3K-Akt inhibition (pan-AKT) chemotherapy	advanced TNBC	OS: 17.7 vs. 18 months, ns	
NCT03337724	3	ipatasertib paclitaxel				placebo paclitaxel			PI3K-Akt inhibition (pan-AKT) chemotherapy	advanced TNBC	PFS (altered TNBC): 7.4 vs. 6.1 months, ns; PFS (not altered TNBC): 7.1 months	
NCT04251533	3	alpelisib nab-p				placebo nab-p			PI3K-Akt inhibition (PI3K) chemotherapy	advanced TNBC; PI3K mut or PTEN loss	PFS: 7.2 vs. 5.6 months, ns; ORR (tumors with PTEN loss): 14.3%	
NCT05954442	3	everolimus investigator’s choice of chemotherapy				placebo investigator’s choice of chemotherapy			PI3K-Akt inhibition (mTOR) chemotherapy	advanced LAR TNBC; PI3K-Akt mut	PFS; ongoing	
NCT02624973	2	olaparib cyclophosphamide							PARPi chemotherapy	untreated	Predictive and prognostic value of mutations in 300 cancer-related genes; ongoing	
NCT03150576	2/3	olaparib paclitaxel and carboplatin				paclitaxel and carboplatin			PARPi chemotherapy	-	pCR: 51% vs. 52%, ns	
NCT04915755	3	niraparib				placebo			PARPi	stage I-III	Number of patients with AEs, change in ECOG performance, change in relevant laboratory parameters, and use of concomitant medications; ongoing	
NCT04799249	3	trilaciclib gemcitabine carboplatin				placebo gemcitabine carboplatin			CDK4/6 chemotherapy chemotherapy	advanced TNBC	OS; ongoing	
**Antibodies**												
NCT04085276	3	toripalimab nab-p				placebo nab-p			immunotherapy (PD-1 blockade)	advanced TNBC	PFS (PD-L1+): 8.4 vs. 5.6 months, significant; PFS (intention-to-treat): 8.4 vs. 6.9 months, significant	
NCT02926196	3	avelumab				observation			immunotherapy (PD-L1 blockade)	high risk, non-mTNBC	DFS: 68.3 vs. 63.4%, ns	
NCT06313463	3	carrellizumab capecitabine				placebo capecitabine			immunotherapy (PD-1 blockade)	stage I-III TNBC	DFS; ongoing	
NCT02499367	2	nivolumab observation/radiation/cyclophosphamide/cisplatin/doxorubicin							immunotherapy (PD-1 blockade) observation/radiation/chemotherapy	advanced TNBC	PFS: 1.9 months	
NCT05078047	3	reduced dose intensity of ICI							immunotherapy	advanced TNBC	PFS; ongoing	
NCT05806060	3	BP102 nab-p + capecitabine/TPC			nab-p + capecitabine/TPC				anti-angiogenic agent	advanced TNBC, basal-like immunosuppressed subtype [42]	PFS; ongoing	
**ADCs**												
NCT05552001	3	SG							TROP2-targeted topoisomerase 1 inhibitor delivery	advanced TNBC	ORR; ongoing	
NCT05382299	3	SG				TPC (paclitaxel/nab-p/gemcitabine/carboplatin)			TROP2-targeted topoisomerase 1 inhibitor delivery chemotherapy	untreated advanced TNBC; untreated and PD-L1- treated with ICI and PD-L1+	PFS; ongoing	
NCT06279364	3	ST				investigator’s choice chemotherapy (paclitaxel/nab-p/capecitabine/eribulin/carboplatin)			TROP2-targeted topoisomerase 1 inhibitor delivery chemotherapy	advanced TNBC	OS, PFS; and ongoing	
NCT05347134	3	ST				eribulin capecitabine gemcitabine vinorelbine			TROP2-targeted topoisomerase 1 inhibitor delivery chemotherapy	advanced TNBC	PFS: 5.8 vs. 1.9 months, significant	
NCT05374512	3	Dato-DXd				investigator’s choice of chemotherapy: paclitaxel/nab-p/carboplatin/capecitabine/eribulin mesylate			TROP2-targeted topoisomerase 1 inhibitor delivery chemotherapy	advanced TNBC	PFS, OS; ongoing	
**Others**												
NCT03562637	3	adagloxad simolenin + OBI-821 SOC (observation, capecitabine, ICI ± and capecitabine)				SOC (observation, capecitabine, and ICI ± capecitabine)			vaccine observation/chemotherapy/immunotherapy	high risk early disease, Globo H+	invasive PFS; ongoing	
**Combinatorial approaches**												
NCT04177108	3	(A) ipatasertib atezolizumab paclitaxel	(B) ipatasertib placebo paclitaxel					(C) placebo placebo paclitaxel	PI3K-Akt inhibition (pan-AKT) immunotherapy (PD-L1 blockade) chemotherapy	advanced TNBC; PD-L1-	PFS (A vs. C): 7.1 vs. 3.7 months, significant; PFS (B vs. C): 5.6 vs. 3.7 months, ns; OS (A vs. C): 15.7 vs. 16.6 months, ns; and OS (B vs. C): 15.3 vs. 16.6 months, ns	
		ipatasertib atezolizumab paclitaxel				ipatasertib placebo paclitaxel			PI3K-Akt inhibition (pan-AKT) immumotherapy (PD-L1 blockade) chemotherapy	advanced TNBC; PD-L1+	PFS: 5.6 vs. 5.7 months, ns; OS: NA vs. 17.2 months, ns	
NCT05760378	3	famitinib camrelizumab nab-p/capecitabine/eribulin mesylate/carboplatin				camrelizumab nab-p/capecitabine/eribulin mesylate/carboplatin			RTK inhibition immunotherapy (PD-1 blockade) chemotherapy	advanced TNBC	PFS; ongoing	
NCT05999149	3	famitinib camrelizumab albumin-paclitaxel, carboplatin				camrelizumab albumin-paclitaxel plus carboplatin			RTK inhibition immunotherapy (PD-1 blockade) chemotherapy	stage II-III TNBC	pCR; ongoing	
NCT05382286	3	SG pembrolizumab				pembrolizumab paclitaxel, nab-p, gemcitabine			TROP2-targeted topoisomerase 1 inhibitor delivery immunotherapy (PD-1 blockade) chemotherapy	previously untreated, advanced TNBC; PD-L1+	PFS; ongoing	
NCT05633654	3	SG pembrolizumab				pembrolizumab capecitabine			TROP2-targeted topoisomerase 1 inhibitor delivery immunotherapy (PD-1 blockade) chemotherapy	pretreated, stage I-III TNBC	invasive DFS; ongoing	
NCT05629585	3	Dato-Dxd durvalumab	Dato-Dxd					pembrolizumab capecitabine	TROP2-targeted topoisomerase 1 inhibitor delivery immunotherapy (PD1/PD-L1 blockade) chemotherapy	stage I-III TNBC	invasive DFS; ongoing	
NCT06112379	3	Dato-DXd durvalumab adjuvant chemotherapy/olaparib in case of residual disease				pembrolizumab chemotherapy			TROP2-targeted topoisomerase 1 inhibitor delivery immunotherapy (PD1/PD-L1 blockade) chemotherapy	previously untreated, stage II-III TNBC	pCR, EFS; ongoing	
NCT06072612	3	SV-BR-1-GM cyclophosphamide peginterferon alfa-2a retifanlimab		TPC			SV-BR-1-GM cyclophosphamide peginterferon alfa-2a		vaccine (cell line secreting GM-CSF injected intradermally) chemotherapy immunoterhapy (interferon) immunotherapy (PD-1 blockade)	advanced TNBC	OS; ongoing	

## Data Availability

No new data were created or analyzed in this study.

## Abbreviations

The following abbreviations are used in this manuscript:

ADC	Antibody-drug conjugates
CDK	Cyclin-dependent kinase
CPS	Combined positive score
CRR	Complete response rate
CT	Chemotherapy
Dato-DXd	Datopotamab deruxtecan
DFS	Disease-free survival
dMMR	DNA mismatch repair deficiency
EFS	Event-free survival
EMA	European Medicines Agency
ESMO	European Society for Medical Oncology
FDA	U.S. Food and Drug Administration
gBRCA1/2	Germline mutations in the breast cancer associated gene 1 or 2
HER2	Human epidermal growth factor receptor 2
HR	Hormone receptors
ICER	Incremental cost-effectiveness ratio
ICI	Immune checkpoint inhibitors
IHC	Immunohistochemistry
ISH	In situ hybridization
LAR	Luminal androgen receptor
LMIC	Low- or middle-income countries
mAb	Monoclonal antibodies
MSI	Microsatellite instability
mTNBC	Metastatic triple-negative breast cancer
mTOR	Mammalian target of rapamycin
nab-p	Nab-paclitaxel
NTRK	Neurotrophic tyrosine receptor kinase
ORR	Overall response rate
OS	Overall survival
PARPi	Poly (ADP-ribose) polymerase (PARP) inhibitors
pCR	Pathological complete response
PD-1	Programmed cell death protein 1
PD-L1	Programmed cell death protein 1 ligand
PFS	Progression-free survival
PI3K	Phosphoinositide 3-kinase
QALY	Quality-adjusted life year
RET	Rearranged during transfection
SG	Sacituzumab govitecan
ST	Sacituzumab tirumotecan
T-DXd	Fam-trastuzumab deruxtecan-nxki
TMB	Tumor mutational burden
TME	Tumor microenvironment
TNBC	Triple-negative breast cancer
TPC	Treatment of physician’s choice
TROP2	Trophoblastic cell-surface antigen-2
TTR	Time to reimbursement
VEGF	Vascular endothelial growth factor
WTP	Willingness-to-pay
